# Real-world analysis of the efficacy and safety of lorlatinib in ALK-positive non-small cell lung cancer patients in China

**DOI:** 10.3389/fonc.2025.1577607

**Published:** 2025-05-01

**Authors:** Guangming Tian, Jun Nie, Ling Dai, Weiheng Hu, Jie Zhang, Di Wu, Xiangjuan Ma, Xiaoling Chen, Sen Han, Jindi Han, Ziran Zhang, Jieran Long, Xinliang Zhao, Jian Fang

**Affiliations:** ^1^ Key Laboratory of Carcinogenesis and Translational Research (Ministry of Education), Thoracic Oncology Department II, Peking University Cancer Hospital & Institute, Beijing, China; ^2^ Department of Medical Genetics, School of Basic Sciences, Peking University, Beijing, China

**Keywords:** NSCLC, lorlatinib, real-world, efficacy, safety, China

## Abstract

**Introduction:**

Lorlatinib, a third-generation ALK inhibitor, has demonstrated strong efficacy in treating advanced ALK-positive NSCLC, though real-world data, particularly from China, are limited. This study evaluates the real-world efficacy and safety of lorlatinib in Chinese patients with advanced ALK-positive NSCLC.

**Materials and methods:**

This retrospective study analyzed 65 patients with advanced ALK-positive NSCLC who received lorlatinib at Peking University Cancer Hospital between September 2017 and August 2024. The study assessed the overall response rate (ORR), progression-free survival (PFS), and safety outcomes, comparing first-line treatment to subsequent treatments after prior ALK inhibitor exposure.

**Results:**

The real-world ORR (rwORR) for all patients was 49.2%, with a real-world disease control rate (rwDCR) of 92.3%. In the first-line treatment group (n=8), lorlatinib showed an ORR of 100%, and no patients experienced progressive disease (PD) during a median follow-up of 9 months. The mPFS for the entire cohort was 37.83 months, with the median OS (mOS) not reached (NR, 95% CI: NR-NR). Patients who had received one prior ALK inhibitor had a mPFS of 49.73 months, while those who had received two or more prior ALK inhibitors had a mPFS of 12.17 months. A statistically significant difference in mOS was found between patients with one prior ALKi and those with two or more prior ALKis (p = 0.032). Lorlatinib demonstrated strong intracranial efficacy, with a 45.2% intracranial ORR in patients with brain metastases. The safety profile was consistent with previous reports, with the most common AEs being hyperlipidemia. However, the incidence of severe AEs was manageable with dose adjustments and supportive treatments.

**Conclusions:**

Lorlatinib demonstrates strong efficacy and manageable safety, especially in first-line treatment of advanced ALK-positive NSCLC, supporting its role as an effective treatment option.

## 1 Introduction

Lung cancer remains the leading cause of cancer-related mortality worldwide and in China, with incidence consistently ranking among the highest for malignant tumors (1, 2). Targeted therapies have significantly prolonged the survival of metastatic non-small cell lung cancer (NSCLC) patients harboring driver gene mutations (3, 4). Anaplastic lymphoma kinase (ALK) gene rearrangements are observed in approximately 2-7% of NSCLC patients and have been established as a key driver mutation in lung cancer (5, 6), particularly prevalent in younger patients and those with no smoking history (7).

Recent advancements have greatly improved treatment options for patients with ALK-positive NSCLC. The PROFILE 1014 study laid the foundation for crizotinib as the first-line treatment for advanced or metastatic ALK-positive NSCLC (8). Subsequently, second-generation ALK inhibitors (ALKis), including ceritinib, alectinib, brigatinib, and ensartinib, have demonstrated superior efficacy and survival benefits compared to crizotinib in several clinical trials (4). More recently, the third-generation ALKi, lorlatinib, which exhibits excellent brain penetration, has shown promising results not only in overcoming ALK resistance mutations but also in first-line treatment settings. In the global, randomized Phase 3 CROWN trial, lorlatinib outperformed crizotinib as first-line therapy for advanced ALK-positive NSCLC patients (9, 10). Both the European Society for Medical Oncology (ESMO) Clinical Practice Guidelines and the National Comprehensive Cancer Network (NCCN) Guidelines for NSCLC recommend second- or third-generation ALKi as the preferred first-line treatment for patients with ALK rearrangements (3, 11). However, the benefits of ALKi are often limited by the emergence of tumoral drug resistance. Recent findings suggest that sequential therapy with ALKi may help overcome resistance and improve overall survival (OS) in advanced or metastatic NSCLC patients (12–17).

The Chinese National Medical Products Administration (NMPA) approved the use of lorlatinib in advanced or metastatic ALK-positive NSCLC patients. However, there remains a lack of comprehensive real-world data regarding its efficacy and safety, particularly within the Chinese patient population. This study aims to evaluate the efficacy and safety profile of lorlatinib, with a specific focus on its therapeutic outcomes in ALK-positive NSCLC patients.

## 2 Materials and methods

### 2.1 Patients

This cohort study includes patients with metastatic NSCLC harboring ALK rearrangements, diagnosed at Peking University Cancer Hospital between September 1, 2013, and July 16, 2024. Eligible patients were aged ≥18 years and had comprehensive medical and treatment records available from the time of their NSCLC diagnosis. All patients enrolled in the study had received lorlatinib for at least 30 days and undergone at least one efficacy assessment.

### 2.2 Study design

Data for this study were retrospectively collected from the patient registry of Peking University Cancer Hospital. Clinical characteristics were extracted or calculated from the database and included diagnostic details (e.g., cancer type and stage), age at diagnosis, gender, smoking history, tumor histopathological type, presence of brain metastases, and disease progression. Information regarding the initial anti-tumor therapy and subsequent treatments was also gathered. Additional clinical details, including medical imaging and blood tests, were updated as part of routine medical checkups. Data collection continued until the patient’s death or the end of the follow-up period (August 30, 2024). Adverse events (AEs) associated with lorlatinib treatment were recorded from the initiation of lorlatinib therapy until the conclusion of follow-up. The primary outcomes, including OS and progression-free survival (PFS), were compared between patients treated with different therapeutic strategies to determine which treatment approach provided the greatest benefit.

### 2.3 Assessments

Tumor staging was reconfirmed according to the 8th Edition of the American Joint Committee on Cancer (AJCC) TNM Classification for Lung Cancer (18). Clinical features and disease progression were assessed by reviewing patient charts and radiographic images. ALK rearrangements were identified using at least one of the following methods: ALK Ventana (D5F3) immunohistochemistry (IHC), fluorescent *in situ* hybridization (FISH) using the Vysis ALK break-apart FISH assay, or next-generation sequencing (NGS). AEs related to lorlatinib treatment were graded by the treating physician according to the Common Terminology Criteria for Adverse Events (CTCAE) version 5.0.

The real-world overall response rate (rwORR) was defined as the proportion of patients achieving a CR or PR. The real-world disease control rate (rwDCR) was defined as the proportion of patients with a CR, PR, or SD. OS was defined as the time from the first dose of lorlatinib to either death or the last follow-up. PFS was defined as the time from the initiation of lorlatinib treatment to either disease progression or death from any cause. Progressive disease(PD) was defined as a ≥20% increase and at least a 5-mm increase in the sum of diameters of target lesions, as per RECIST, version 1.1 (19).

### 2.4 Ethical statement

All procedures were conducted in accordance with relevant guidelines and regulations. The study was approved by the Ethics Committee of Peking University Cancer Hospital (Approval No. 2021KT21). Clinical information was provided in a de-identified format. The study was performed in accordance with the Declaration of Helsinki (as revised in 2013).

Individual consent for this retrospective analysis was waived by the Ethics Committee.

### 2.5 Statistical analysis

The Kaplan-Meier method was used to estimate the median PFS for each treatment group, with 95% confidence intervals. The assessments between treatment groups regarding survival outcomes were performed using a stratified log-rank test at a 5% significance level. All statistical tests were two-sided, and a P-value < 0.05 was considered statistically significant. Statistical analyses were conducted using SPSS software (version 26, IBM Corp., Armonk, NY, USA).

## 3 Results

### 3.1 Patient characteristics

A total of 322 patients diagnosed with metastatic NSCLC harboring ALK rearrangements, confirmed by histopathological and imaging examinations, were identified between September 1, 2013, and August 30, 2024, with median follow-up for 52.8 months. Of these, 68 patients received lorlatinib treatment between September 1, 2017, and August 30, 2024. Three patients were excluded from the analysis due to discontinuing lorlatinib within one month, leaving 65 patients in the final data analysis. Among these, 8 received lorlatinib as their first ALKi, 25 had previously received one type of ALKi, 20 had received two other ALKi, and 12 had received more than two types of ALKi. The median follow-up for PFS and OS was 9 months. The histological classifications of these NSCLC patients were adenocarcinoma (n=61), squamous cell carcinoma (n=1) and atypical carcinoid (n=1), respectively, while 2 NSCLC patients were lack of classification information. A total of 50 patients received only one test method for detecting ALK rearrangement, including 23 patients with IHC, 26 patients with NGS and 1 patient with FISH, all patients got positive results. There were 14 patients who received two detection methods, with 11 using both IHC and NGS (IHC+/NGS+, n=9; IHC+/NGS-, n=2), and 3 using both IHC and FISH (IHC+/FISH+, n=2; IHC+/FISH-, n=1). Additionally, there was 1 patient who underwent IHC, NGS, and FISH detection (IHC+/FISH-/NGS+). Patient characteristics are summarized in **Table 1**.

**Table 1:** Patients Demographics.

Characteristics	N=65
Gender	N (%)
Female	40 (61.54)
Male	25 (38.46)
Age at initial diagnosis	Years
Median	51
Range	26-69
Age at first dose of Lorlatinib	Years
Median	56
Range	27-76
Smoking history	N (%)
Current smoker	2 (3.08)
Former smoker	13 (20.00)
Never	47 (72.31)
Unknown	3 (4.62)
ECOG performance status	N (%)
0	31 (47.69)
1	26 (40.00)
2	6 (9.23)
3	2 (3.08)
Histological classification	N (%)
Adenocarcinoma	61 (93.85)
Squamous cell carcinoma	1 (1.54)
Atypical carcinoid	1 (1.54)
Unknown	2 (3.08)
Metastases at first dose of Lorlatinib	N (%)
Lung	33 (50.77)
Brain	42 (64.62)
Leptomeninges	6 (9.23)
Bone	29 (44.62)
Pleura/pericadium	31 (47.69)
Adrenal gland	6 (9.23)
Liver	12 (18.46)
Other^a^	4 (6.15)
Prior ALKi	N (%)
None	8 (12.31)
Crizotinib	34 (52.31)
Alectinib	33 (50.77)
Brigatinib	13 (20.00)
Ceritinib	10 (15.38)
Ensartinib	18 (27.69)

a Abdominal mass, n=2. Pancreatic metastasis, n=2.

### 3.2 Efficacy of lorlatinib

In the cohort of 65 patients enrolled in our study, the therapeutic efficacy of lorlatinib was evaluated as follows: complete response (CR) was observed in 3 patients (4.6%), partial response (PR) in 29 patients (44.6%), stable disease (SD) in 28 patients (43.1%), and progressive disease (PD) in 5 patients (7.7%). The real-world overall response rate (rwORR) was 49.2%, and the real-world disease control rate (rwDCR) was 92.3%.

Among the 42 patients with brain metastases, 6 patients received lorlatinib as first-line therapy and had not undergone cranial radiotherapy either before or during lorlatinib treatment. Among the remaining 36 patients who received lorlatinib as second-line or later therapy, 19 patients had received cranial radiotherapy for progressive brain metastases prior to initiating lorlatinib, 5 patients received cranial radiotherapy during the lorlatinb treatment period. Of the 42 patients who had brain metastases before receiving lorlatinib, 0 patients (0.0%) got CR, 21 patients (50.0%) got PR, 16 patients (38.1%) got SD, and 5 patients (7.7%) got PD. The rwORR was 50.0%, and the rwDCR was 90.5%. Within the group of patients with brain metastases, the intracranial response showed that 9 patients (21.4%) got CR, with an intracranial rwORR of 45.2% and an intracranial rwDCR of 92.9%. Extracranial lesions had an rwORR of 36.6% and a rwDCR of 92.7%.

Among the 23 patients without brain metastases before lorlatinib treatment, 3 patients (13.0%) got CR, 8 patients (34.8%) got PR, and 12 patients (52.2%) got SD. There was no primary drug resistance (no patients had PD as the best response). The rwORR in this group was 47.8%, and the rwDCR was 100%.

No significant difference was found in the ORR and DCR of lorlatinib between patients with brain metastases and those without brain metastases before treatment (as shown in **Table 2**).

**Table 2:** Tumor assessment of lorlatinib.

		Overall	Brain metastasis			Non-brain metastasis (%)	p
		N (%)	Total	Intracranial (%)	Extracranial (%)		BM vs Non-BM
Total		65 (100.0)	42 (100.0)	42 (100.0)	42 (100.0)	23 (100.0)	
Best response of lorlatinib	CR	3 (4.6)	0(0.0)	9 (21.4)	1 (2.4)	3 (13.0)	
	PR	29 (44.6)	21(50.0)	10 (23.8)	14 (33.3)	8 (34.8)	
	SD	28 (43.1)	16(38.1)	20 (47.6)	24 (57.1)	12 (52.2)	
	PD	5 (7.7)	5 (11.9)	3 (7.1)	3 (7.1)	0 (0.0)	
rwORR		32 (49.2)	21(50.0)	19 (45.2)	15 (35.7)	11 (47.8)	χ^2^ = 0.028, p=0.867
rwDCR		60 (92.3)	38 (90.5)	39 (92.9)	39 (92.9)	23 (100.0)	χ^2^ = 2.966, p=0.152

ECOG, Eastern Cooperative Oncology Group.

CR, complete response; PR, partial response; SD, stable disease; PD, progressive disease; rwORR, real-world overall response rate; rwDCR, real-world disease control rate.

The analysis of the 65 patients treated with lorlatinib revealed a median PFS (mPFS) of 37.83 months, with the median OS (mOS) not reached (NR, 95% CI: NR-NR). The efficacy of lorlatinib based on prior ALKi treatment history is summarized in **Table 3**. In the ALKi-naive cohort, the ORR was 100%, with a statistically significant difference in ORR observed between patients receiving lorlatinib as first-line therapy and those with prior ALKi exposure (χ² = 11.15, p = 0.004). The DCR for ALKi-naive patients was 100%, although no significant difference was found in DCR between the first-line lorlatinib group and those previously treated with other ALKi(s) (χ² = 5.59, p = 0.061). In this cohort, both mPFS and mOS were not reached.Among patients who had previously received only one ALKi (n = 25, 38.5%), the rwORR was 48.0%, and the rwDCR was 100%. The mPFS for this group was 49.73 months (95% CI: NR-NR), and mOS was not reached (95% CI: NR-NR). For those who had been treated with two or more prior ALKi agents (n = 32, 49.2%), the rwORR was 34.4%, the rwDCR was 84.4%, and mPFS was 12.17 months (95% CI: 3.19-21.15). The mOS in this cohort was not reached (95% CI: NR-NR).

**Table 3:** Treatment effectiveness of lorlatinib.

Prior ALKi treatment	N(%)	Best Response				rwORR (%)	rwDCR (%)	mPFS (months) 95%CI	mOS(lor) (months) 95%CI
		CR	PR	SD	PD				
Overall	65 (100.0)	3	29	28	5	50.8	92.3	37.83 (2.75-72.91)	NR
ALKi naive	8 (12.3)	1	7	0	0	100.0	100.0	NR	NR
1 prior ALKi	25 (38.5)	1	12	12	0	52.0	100.0	49.73 (NR-NR)	NR
≥2 prior ALKis	32 (49.2)	1	10	16	5	34.4	84.4	12.17 (3.19-21.15)	NR

CR, complete response; PR, partial response; SD, stable disease; PD, progressive disease; rwORR, real-world overall response rate; rwDCR, real-world disease control rate.

### 3.3 Survival outcome of lorlatinib

**Figure 1:**
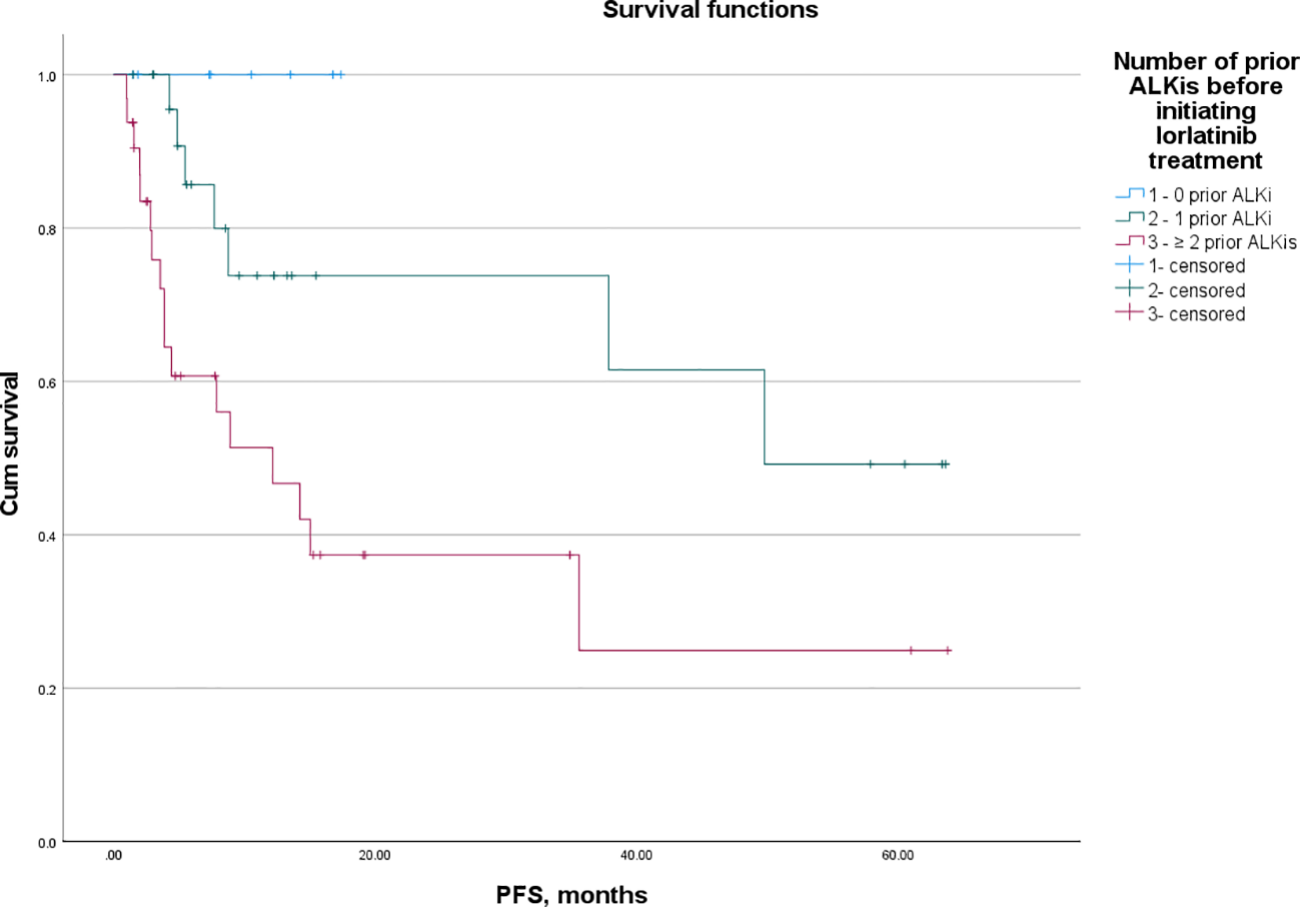
PFS of lorlatinib in patients that received 0, 1, and ≥ 2 prior ALKis before initial lorlatinib.

**Figure 1** illustrates the PFS and OS of lorlatinib in patients who received 0, 1, and ≥2 prior ALKis before initiating lorlatinib treatment. Among patients treated with lorlatinib as a first-line therapy (n = 8), the median follow-up for PFS was 9 months, with no PD observed. The mPFS was not reached (95% CI: NR-NR). In patients who had received one prior ALKi before lorlatinib initiation, the mPFS was 49.73 months (95% CI: NR-NR). In contrast, patients who had previously received two or more ALKis had a mPFS of 12.17 months (95% CI: 3.19-21.15).There was no significant difference in mPFS between first-line lorlatinib users and those who had received one prior ALKi (p = 0.224). However, first-line lorlatinib users experienced significantly longer mPFS compared to those with two or more prior ALKis (p = 0.035). Additionally, patients who had received one prior ALKi had significantly longer mPFS than those who had received two or more prior ALKis (p = 0.018).Regarding mOS, no significant differences were observed between first-line lorlatinib users and those who had received one prior ALKi (p = 0.602) or two or more prior ALKis (p = 0.098). However, a statistically significant difference in mOS was found between patients with one prior ALKi and those with two or more prior ALKis (p = 0.032).

**Figure 2:**
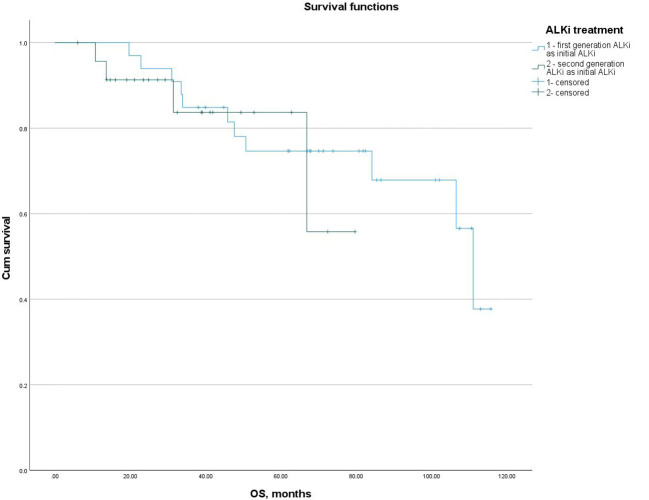
mOS from the initiation of the first ALKi in patients who received first- or second-generation ALKis as their initial ALKi.

For patients who did not receive lorlatinib as their initial ALKi, the mOS from the initiation of the first ALKi was 111.00 months (95% CI: 80.22-141.78), with a 5-year survival rate of 80.7%. No significant difference in mOS was observed between patients who received first- or second-generation ALKis as their initial ALKi (111.00 vs NR, p = 0.624). The 5-year survival rates for these groups were 75.8% and 87.5%, respectively, with no significant difference (p = 0.326) (**Figure 2**).

Disease progression was observed in 24 of the 65 patients. The progression patterns included intracranial progression (n = 10), extracranial progression (n = 11), and both intracranial and extracranial progression (n = 3).Among the 24 patients who experienced PD on lorlatinib, 12 continued lorlatinib therapy post-PD. Of these, 8 patients continued lorlatinib monotherapy, and 4 received lorlatinib in combination with anlotinib. In contrast, the other 12 patients who discontinued lorlatinib treatment had varying therapeutic approaches: 6 received best supportive care, 3 were switched to another ALKi, 2 underwent chemotherapy, and 1 received another ALKi after progressing on chemotherapy.

Statistical analysis revealed that the mOS in patients who continued lorlatinib therapy after PD was 57.17 months (95% CI: 3.24-111.10), compared to just 4.07 months (95% CI: 0.00-35.78) in the group that discontinued lorlatinib after PD. The difference between these two groups was statistically significant (p = 0.029).

### 3.4 Safty of lorlatinib

Among the 65 patients in the safety analysis set, the most frequently reported AEs were hyperlipidemia, including hypercholesterolemia (n = 53, 81.5%) and hypertriglyceridemia (n = 47, 72.3%). Other AEs with an incidence of ≥10% included elevated transaminases (n = 10, 15.4%), neurocognitive AEs (n = 10, 15.4%), hyperglycemia (n = 8, 12.3%), and edema (n = 7, 10.8%).

Grade 3 or higher AEs were observed in the following patients: hypercholesterolemia (n = 10, 15.4%), hypertriglyceridemia (n = 13, 18.5%), neurocognitive AEs (n = 5, 7.7%), hyperglycemia (n = 2, 3.1%), hypertension (n = 1, 1.5%), and hyperbilirubinemia (n = 1, 1.5%) (**Table 4**). Specifically, among the 8 patients receiving first-line lorlatinib treatment, Grade 3 or higher AEs included hypertriglyceridemia (n = 1, 12.5%) and hyperglycemia (n = 1, 12.5%).

**Table 4:** Adverse events in lorlatinib-treated patients (n = 65).

N=65	Total	Grade 1	Grade 2	Grade 3	Grade 4	Unknown
Neurocognitive Adverse Events	10 (15.4%)	3 (4.6%)	2 (3.1%)	4 (6.2%)	1 (1.5%)	–
Hypercholesteremia	53 (81.5%)	16 (24.6%)	21 (32.3%)	6 (9.2%)	4 (6.2%)	6 (9.2%)
Hypertriglyceridemia	47 (72.3%)	12 (18.5%)	17 (26.2%)	7 (10.8%)	5 (7.7%)	6 (9.2%)
Transaminase elevated	10 (15.4%)	6 (9.2%)	3 (4.6%)	–	1 (1.5%)	–
Hyperglycemia	8 (12.3%)	3 (4.6%)	2 (3.1%)	2 (3.1%)	–	1 (1.5%)
Edema	7 (10.8%)	5 (7.7%)	1 (1.5%)	–	–	1 (1.5%)
CK-MB elevated	2 (3.1%)	2 (3.1%)	–	–	–	–
Body weight gain	5 (7.7%)	3 (4.6%)	2 (3.1%)	–	–	–
Other	4 (6.2%)	2 (3.1%)	–	2 (3.1%)	–	–

Other adverse events: hypertension, grade 3, n=1; hyperbilirubinemia, grade 3, n=1; creatine kinase elevated, grade1, n=2.

Neurocognitive AEs were reported in 10 of the 65 patients, resulting in an incidence rate of 15.4%. Cognitive function changes were observed in 5 patients (50.0%), mood changes in 5 patients (50.0%), and 2 of these patients (20.0%) also experienced language function changes. No significant differences were found between patients with and without neurocognitive AEs regarding brain metastasis status, number of prior treatment regimens, history of brain radiotherapy, age, or gender (p > 0.05).

A total of 11 patients required lorlatinib suspension due to Grade 3 or higher AEs, including neurocognitive AEs (n = 5), hyperlipidemia (n = 3), elevated transaminases (n = 1), and hyperglycemia (n = 2). All adverse events recovered to ≤ Grade 1 within 2 weeks following the suspension of lorlatinib. Five patients reduced the lorlatinib dose to 75 mg once daily, while 6 patients resumed the original dose with supportive treatments, such as lipid-lowering medications. Notably, no AEs of ≥ Grade 3 occurred again after these adjustments.

## 4 Discussion

Lorlatinib is a third-generation ALKi with a unique macrocyclic structure, potent against both wild-type ALK rearrangements and clinically reported ALK kinase domain mutations (20). It has demonstrated high activity against various EML4-ALK variants (including v1 and v3) (21), as well as a broad spectrum of acquired single ALK point mutations (including G1202R), with lower IC50 values compared to first- and second-generation ALKi (22).

This study is a single-center, retrospective analysis aimed at exploring the real-world efficacy and safety of lorlatinib in the treatment of ALK-positive advanced NSCLC. In a global phase 2 study (23), the ORR in 30 ALKi-naive patients with ALK-positive advanced NSCLC was 90.0%. In 198 patients who had received at least one prior ALKi, the ORR was 47.0%, and in 111 patients who had received at least two prior ALKis, the ORR was 38.7%. In our study, the ORR for ALKi-naive patients reached 100%, though the sample size was small (n=8), which may introduce bias. For patients who had received prior treatment, the ORR was consistent with the findings from the global study.

Brain metastasis is a common site of metastasis in ALK-positive advanced NSCLC and is one of the most frequent sites of progression in drug resistance. Up to 70% of patients with ALK rearrangement with NSCLC develop brain metastases, making ALK gene rearrangements a prime target for TKIs that can penetrate the blood-brain barrier (24). In this study, no significant differences were observed in the ORR and DCR between patients with and without brain metastasis prior to treatment, suggesting that the brain metastasis does not affect the efficacy of lorlatinib. This finding aligns with the efficaciously penetration across the blood-brain barrier of lorlatinib, and showed substantial intracranial activity in patients with ALK-positive NSCLC. Among the 65 patients in this study, 64.6% (42/65) had brain metastases before treatment, and the intracranial ORR was 45.2%, with a DCR of 92.9%. The findings from different real-world studies vary slightly. An international multicenter real-world study analyzed data from 76 ALK-positive NSCLC patients treated with second-line or later lorlatinib, including 52 patients with brain metastasis.In that study, the intracranial ORR was 35%, and the DCR was 87% (25). The global real-world study GLASS, which analyzed the efficacy of lorlatinib in 106 ALK-positive advanced lung adenocarcinoma patients, showed an ORR of 62% and a DCR of 88% in patients with brain metastasis (26). A Korean population based real-world study reported an intracranial ORR of 60% in ALK-positive advanced NSCLC patients with brain metastasis treated with lorlatinib (20). The differences in ORR may be attributed to the proportion of first-line patients and the varying clinical judgment evaluation criteria. However, the relatively consistent DCR across studies further emphasizes that lorlatinib’s ability of central nervous system penetration.

ALKis have been the standard first-line treatment for advanced NSCLC patients with ALK rearrangements for over a decade (10, 27–30). In the CROWN trial, the median duration of follow-up for PFS was 60.2 months for the first-line lorlatinib group. The mPFS according to investigator assessment was not reached (HR 0.19, 95% CI: 64.3 - NR), and the estimated proportion of patients alive without disease progression was 60% at 5 years (10). In a phase 2 study involving 109 Chinese patients, the mPFS (95% CI) per independent central review was 26.3 months (95% CI: 16.6-35.9) in cohort 1 (n = 67, lorlatinib following disease progression after receiving crizotinib as the only ALK inhibitor) and 5.6 months (95% CI: 2.9-12.4) in cohort 2 (n = 42, lorlatinib after receiving another ALK inhibitor, with or without prior crizotinib), respectively. The median duration of follow-up for OS was 36.4 months and 37.5 months, and the mOS was NR (95% CI: NR-NR) and 21.9 months (95% CI: 11.9-NR), respectively. The ORR was 79.1% (95% CI: 67.4-88.1%) in cohort 1 and 47.6% (95% CI: 32.0-63.6%) in cohort 2, while the intracranial ORR was 83.8% (95% CI: 68.0-93.8%) in cohort 1 and 50.0% (95% CI: 28.2-71.8%) in cohort 2 (31). In our study, the median follow-up time for first-line lorlatinib was 9 months, which is significantly shorter than the median follow-up time for PFS in first-line lorlatinib reported in other clinical trials. As a result, the PFS data in our study are immature, and the extension of follow-up is required. The lack of significant difference in mPFS between the first-line and second-line lorlatinib treatment groups may also be attributed to the short follow-up period for the first-line treatment group. As the number of prior ALKi treatments increases, the ORR for lorlatinib gradually decreases. The mPFS in the ≥2-line treatment group was significantly shorter compared to the first- and second-line treatment groups, consistent with findings from phase 2 studies and other real-world studies. A potential mechanism for this observed decline in lorlatinib efficacy may be the increased likelihood of compound mutations in the target gene after multiple ALKi treatments, which could lead to reduced efficacy of lorlatinib (32).

We observed that some patients continued lorlatinib treatment after disease progression. The potential rationale for this could be oligoprogression, as well as limited access to other treatment options capable of overcoming lorlatinib resistance. Physicians may opt to add other therapies to lorlatinib as an exploratory regimen to potentially extend OS. While continued lorlatinib treatment after progression was associated with OS benefits, the limit sample size limits our ability to control for and stratify potential confounding factors. These findings suggest a potential benefit that warrants further investigation in confirmatory studies.

The safety profile of lorlatinib in our study was consistent with that observed in phase 2 and phase 3 clinical trials, with no new safety signals identified (10, 31). The most common lorlatinib-related AE in our cohort was hyperlipidemia, and nearly all patients with this condition required ongoing treatment with lipid-lowering medications. Notably, neurocognitive AEs were reported in 15.4% (10/65) of patients in our cohort, which was lower than the incidence observed in global clinical trials but higher than in Chinese clinical trials (10, 31, 33). In a phase 1/2 global trial (NCT01970865), a subgroup analysis of lorlatinib-related AEs in Asian versus non-Asian patients revealed non-Asian patients had higher incidences of neurocognitive effects, and some other AEs, such as fatigue, increased amylase, and anemia (33). In a multicenter phase 2 study conducted in China (NCT03909971), neurocognitive AEs were reported in only 8 patients (7.3%), including cognitive (2.8%), mood (2.8%), psychotic (0.9%), and speech (0.9%) disturbances (31). Generally, the inconsistency in the incidence of neurocognitive AEs in our study may be attributed to mild symptoms that were not captured or recorded in outpatient clinical records. Additionally, cultural factors may make it difficult for many local individuals to recognize neurocognitive disorders. More detailed medical history documentation and greater awareness of neurocognitive AEs could help in the timely identification, evaluation, and intervention of these events.

The limitations of this study include the relatively small sample size, which is a result of the rarity of the indication of ALK rearrangement, poor lorlatinib accessibility, potentially introducing statistical bias. As such, the interpretation of the stratified results should be considered exploratory. Additionally, the follow-up period may not have been long enough to fully assess the long-term efficacy and safety of lorlatinib. Furthermore, as the study was conducted at a single center, the generalizability of the results may be limited. Future studies with larger sample sizes and longer follow-up periods are needed to further validate our findings.

## 5 Conclusion

In conclusion, our single - center, retrospective analysis provides valuable insights into the real - world efficacy, survival, and safety of lorlatinib in patients with advanced NSCLC harboring ALK rearrangements. In ALKi - naive patients, the ORR reached 100% (small sample size). ORR in pre - treated patients was consistent with global studies. Brain metastasis didn’t affect lorlatinib’s efficacy. The PFS data in our first - line lorlatinib group were immature due to short follow - up. The safety profile was consistent with clinical trials. The findings may have important implications for clinical practice. However, further studies with larger sample sizes and longer follow-up periods are needed to confirm our results and explore the optimal use of lorlatinib in the management of NSCLC.

## Data Availability

The original contributions presented in the study are included in the article, further inquiries can be directed to the corresponding authors.
